# Peroxisome proliferator-activated receptor gamma and its natural agonists in the treatment of kidney diseases

**DOI:** 10.3389/fphar.2022.991059

**Published:** 2022-10-21

**Authors:** Vinesh Sharma, Vikram Patial

**Affiliations:** ^1^ Pharmacology and Toxicology Laboratory, Dietetics & Nutrition Technology Division, CSIR-Institute of Himalayan Bioresource Technology, Palampur, HP, India; ^2^ Academy of Scientific and Innovative Research (AcSIR), Ghaziabad, UP, India

**Keywords:** kidney diseases, natural agonists, PPAR-γ, synthetic agonists, pathophysiology

## Abstract

Kidney disease is one of the leading non-communicable diseases related to tremendous health and economic burden globally. Diabetes, hypertension, obesity and cardiovascular conditions are the major risk factors for kidney disease, followed by infections, toxicity and autoimmune causes. The peroxisome proliferator-activated receptor gamma (PPAR-γ) is a ligand-activated nuclear receptor that plays an essential role in kidney physiology and disease. The synthetic agonists of PPAR-γ shows a therapeutic effect in various kidney conditions; however, the associated side effect restricts their use. Therefore, there is an increasing interest in exploring natural products with PPARγ-activating potential, which can be a promising solution to developing effective and safe treatment of kidney diseases. In this review, we have discussed the role of PPAR-γ in the pathophysiology of kidney disease and the potential of natural PPAR-γ agonists in treating various kidney diseases, including acute kidney injury, diabetic kidney disease, obesity-induced nephropathy, hypertension nephropathy and IgA nephropathy. PPAR-γ is a potential target for the natural PPAR-γ agonists against kidney disease; however, more studies are required in this direction.

## Introduction

Renal disease is one of the most important non-communicable diseases contributing to a global economic burden. There are multiple causes of renal diseases, but the major risk factors are diabetes, hypertension, obesity and cardiovascular conditions (Luyckx et al., 2018). A WHO report of the year 2015 revealed a 32% increase in mortality due to kidney failure since 2005. In addition, approximately 1.7 million people worldwide die from acute kidney disease, while 5–10 million die from kidney diseases (Luyckx et al., 2018). In European countries, the number of cases of end-stage kidney disease is rising at a rate of 5%–8% annually. The developing countries spend almost 2–3% of their health care budget on treating ESKD; however, the number of patients receiving such treatment constitutes 0.03% of the total community (Wetmore and Collins, 2016; Ng et al., 2020). In many countries, due to the limitation of renal replacement services, it is estimated that around 2.3–7.1 million adults have died prematurely due to lack of treatment. In addition, the estimated number of people undergoing renal replacement therapy by the year 2030 is projected double to 5.4 million. The most effective methods to overcome the growing global burden of kidney disease are early identification, specific drug availability and risk management (Bikbov et al., 2020).

The peroxisome proliferator-activated receptor gamma (PPAR-γ) is a ligand-activated nuclear receptor that regulates the transactivation or transrepression of specific genes involved in various biological functions. PPAR-γ participates in the general transcriptional control of different cellular processes, including glucose and lipid metabolism, cell differentiation, adipocyte differentiation and immunity (Corton et al., 2000; Croasdell et al., 2015). PPAR-γ is abundantly expressed in adipose tissue with a primary role in adipogenesis. However, recently lower expression of PPAR-γ was identified in the kidney, adrenal, intestine, heart, lung, liver, brain, and vasculature (Grabner et al., 2021). A close interplay of PPAR-γ was studied in the pathogenesis of renal diseases. The pharmacological activation of PPAR-γ facilitates the regulation of glucose metabolism, free fatty acid and liver gluconeogenesis (Kiss-Tóth and Rőszer, 2009). Several selective modulators of PPAR-γ showed therapeutic effects in various conditions (Sun et al., 2016). There is an increasing interest in natural products for medicinal purposes due to their multi-targeted efficacy and relatively fewer side effects. Further, continuous efforts are made to explore the natural PPAR-γ agonists treating renal conditions. So, we have reviewed the different natural products used to manage renal diseases, including acute kidney injury, diabetic nephropathy, obesity-related nephropathy, hypertension-induced nephropathy, IgA nephropathy and renal cancer.

## PPAR-γ transcription and role in renal pathophysiology

**FIGURE 1:**
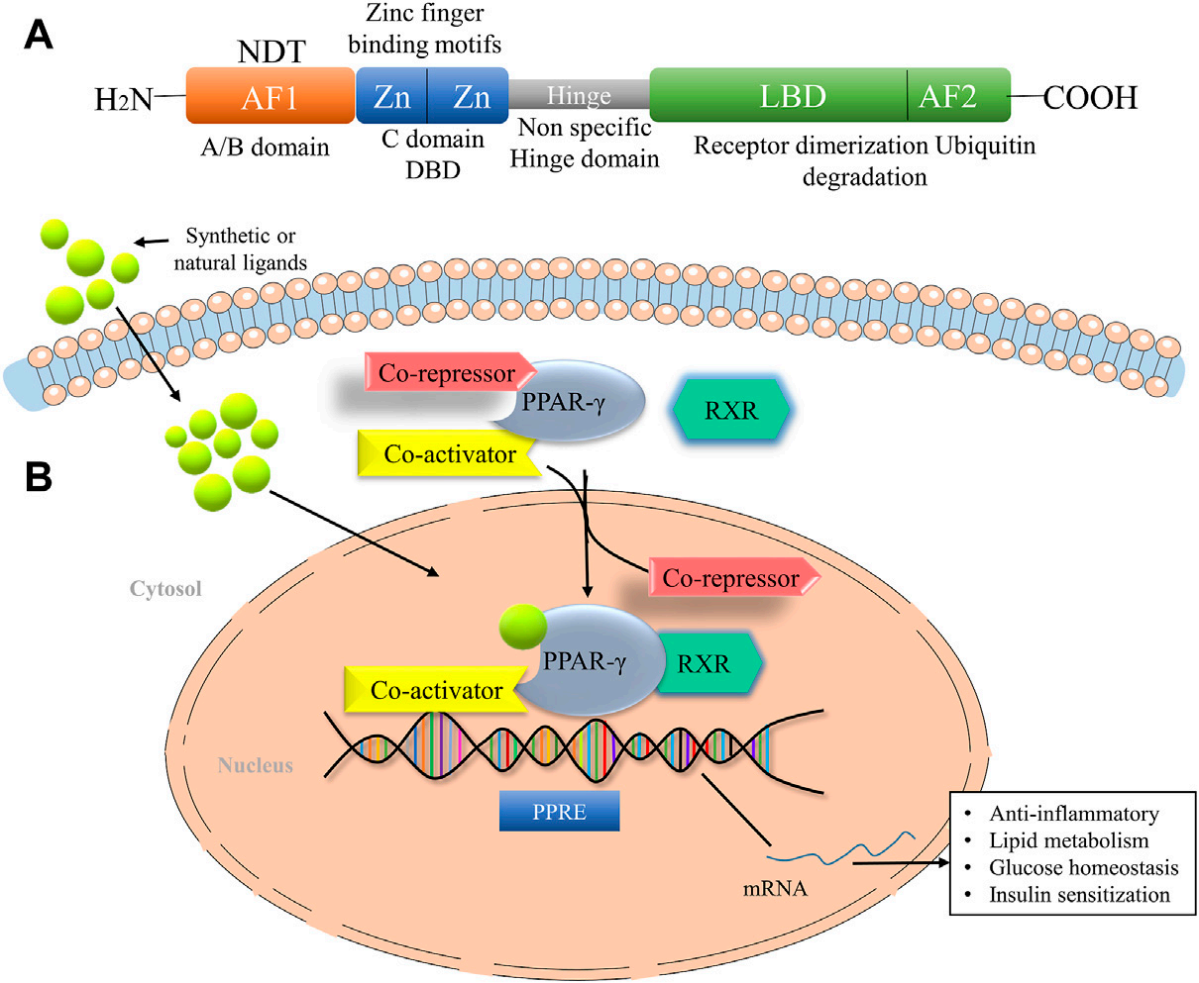
The domain structure of PPAR-γ and their mode of action. **(A)** Domain Structure of PPAR-γ facilitates the DNA binding at the DNA-binding domain (DBD). The ligand-binding domain (LBD) senses the agonists through the ligand-binding pocket. **(B)** PPAR-γ ligand binding to heterodimer consist of RXR, PPAR-γ, and corepressor promote the activation of PPAR-γ transcription through binding with coactivator. AF1, Activation function 1, AF2, Activation function 1, NTD, N-terminal domain, LBD, Ligand binding domain, RXR, Retinoid X receptor, NF-кB, Nuclear factor kappa-light-chain-enhancer of activated B cells, STAT, Signal transducer and activator of transcription.

PPAR-γ is a ligand-dependent transcription factor and member of the nuclear receptor superfamily, which is regulated by the direct binding of lipid metabolites, thyroid and steroid hormones, vitamins and xenobiotics. It is mainly located in the nucleoplasm and the vesicles. The human PPAR-γ gene is ∼ 100 kb long with nine exons and is located in the p25 region of the chromosome (Zhou et al., 2002; Katoch, et al., 2022). PPAR gene is located in different organs and involved in various pharmacological processes, which include fatty acid metabolism, glucose homeostasis and insulin sensitization, regulation of cell proliferation, tissue damage, tumour repair and inflammation (Costa et al., 2010). The PPAR-γ structure consists of an N-terminal domain for ligand-independent transactivation function (AF1), a DNA binding domain with two protein motifs and a C-terminal ligand-binding domain (LBD) for ligand-dependent activation (AF2) (Lefterova et al., 2014) (Figure 1A). It further consists of two isoforms, PPAR-γ1 and PPAR-γ2. The presence of additional 30 amino acids in the N terminal domain of the PPAR-γ2 isoform makes it a more potent transcription activator than PPAR-γ1 and crucial in the low concentrations of ligands (Costa et al., 2010). PPAR-γ2 is most prominently expressed in the adipose tissue, whereas many other tissues express PPAR-γ1 at a low level (Li et al., 2016). Multiple mechanisms control the transcription of PPAR target genes in a target cell or tissue. After ligand stimulation, PPAR-γ translocates to the nucleus and heterodimerizes with another member of the family, retinoid X receptors (RXR). RXR, in combination with PPAR-γ, act as a master regulator of many critical pathways; however, do not function independently. The heterodimer complex binds to specific DNA sequences known as peroxisome proliferator hormone response elements (PPREs) of the promoter region of target genes (Ma et al., 2018). It consists of indirect repeats of the sequence AGGTCA, which are further distinguished by a single base pair (Figure 1B).

**FIGURE 2:**
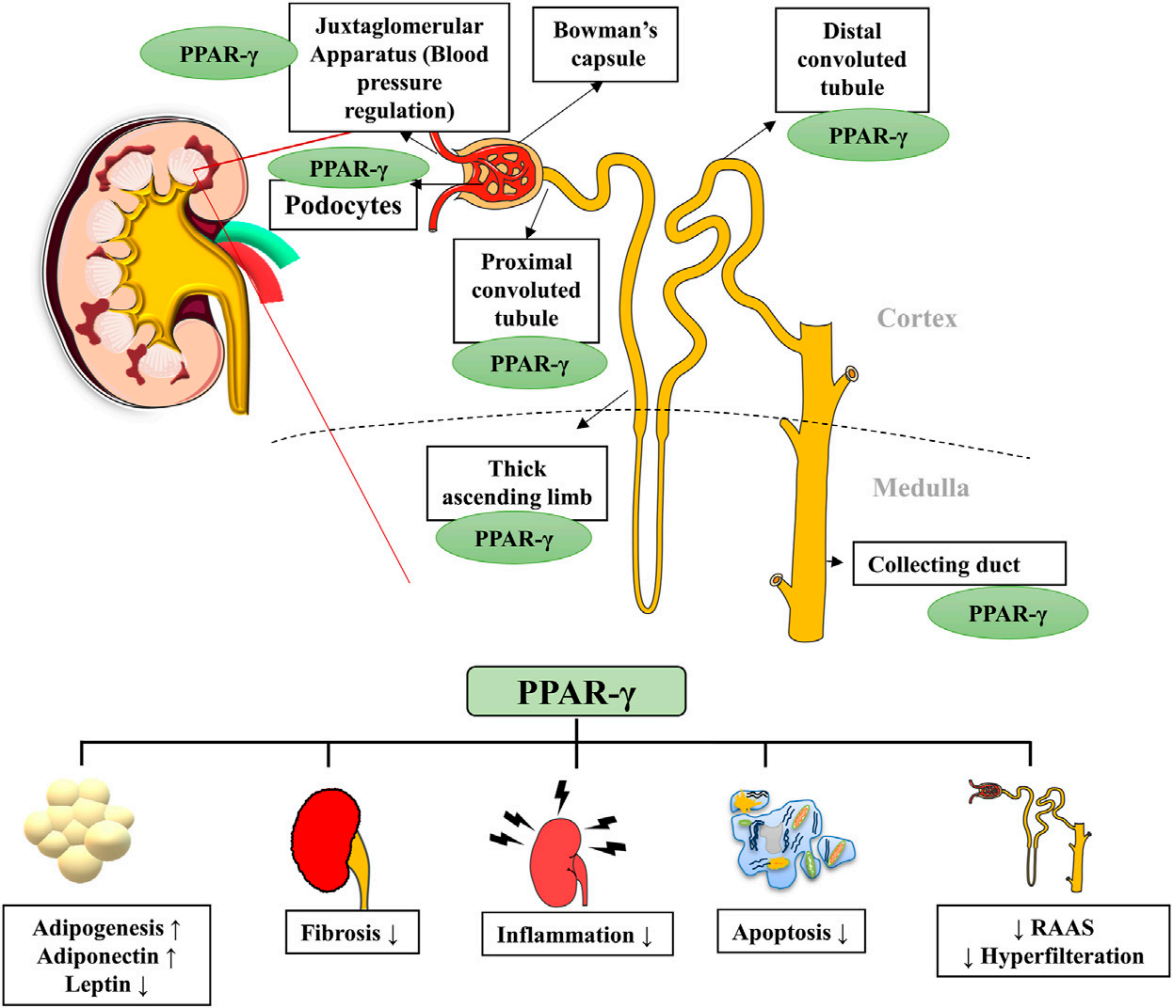
PPAR-γ presence in various parts of the nephron and its major functions. PPAR-γ is expressed in different parts of the nephron, such as bowman’s capsule, podocytes, proximal convoluted tubules, distal convoluted tubules and collecting ducts. PPAR-γ has a role in kidney inflammation, lipid metabolism, hypertension, fibrosis and apoptosis. RAAS, Renin-angiotensin-aldosterone system.

**FIGURE 3:**
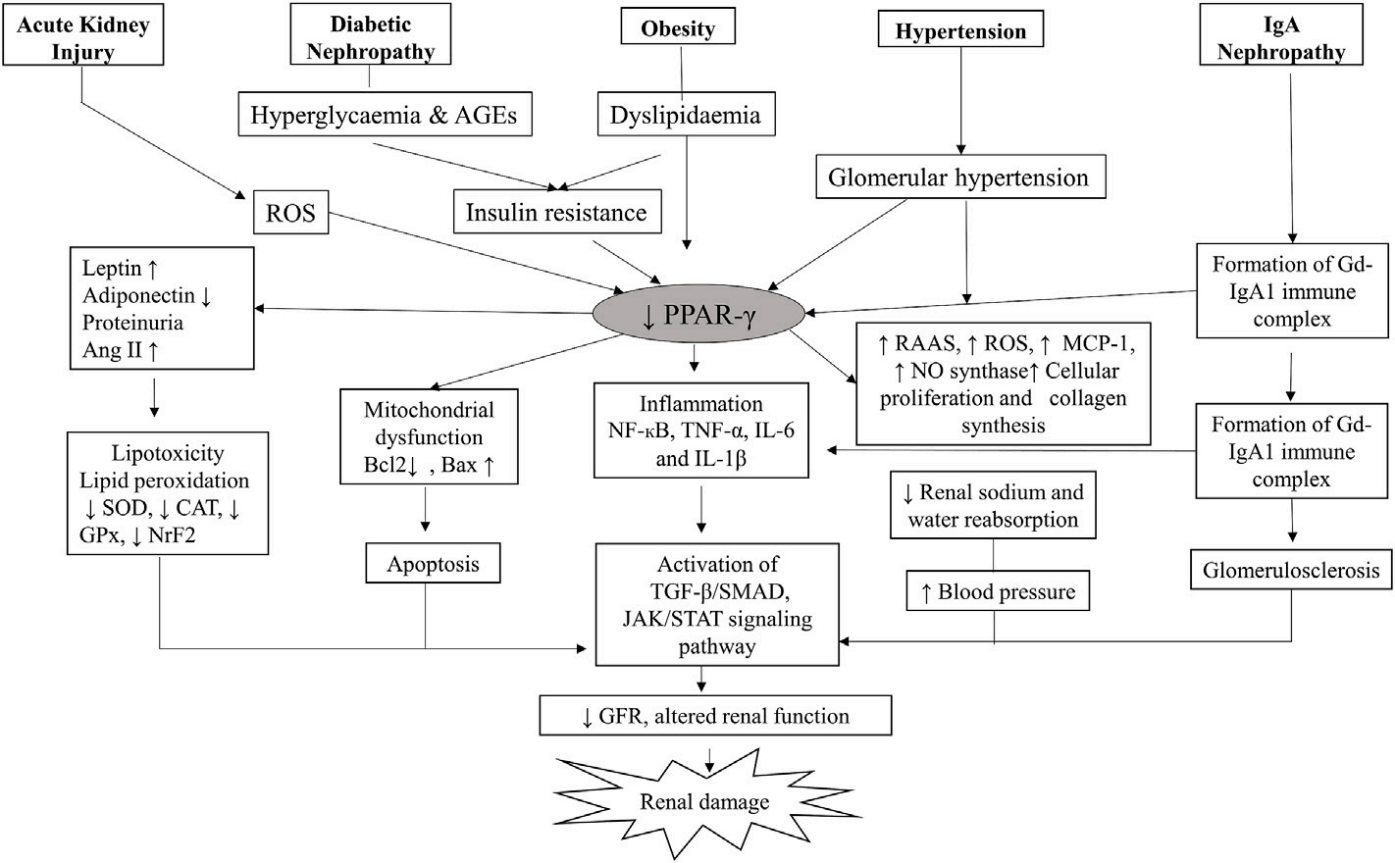
Role of PPAR-γ in the pathophysiology of kidney diseases. The suppression of PPAR-γ in disease conditions further activates the inflammatory and fibrogenic factors leading to kidney damage. AGE, Advanced glycation end products; BAX, BCL2-associated X protein; BCL-2, B-cell leukemia/lymphoma 2; CAT, Catalase; GPx, Glutathione peroxidase; GFR, Glomerular filtration rate; IL-6, Interleukin-6; IL-1β, Interleukin-1 β, IgA1, Immunoglobulins A1; MCP1, Monocyte chemoattractant protein-1; NFκB, Nuclear factor-kappa B light-chain-enhancer of activated B cells; NO, Nitric oxide; NrF2, Nuclear factor-erythroid factor 2-related factor 2; ROS, Reactive oxygen species; RAAS, Renin-Angiotensin-Aldosterone System; SOD, Superoxide dismutase; STAT, Signal transducer and activator of transcription; TGFβ, Transforming growth factor-beta; TNFα, Tumor necrosis factor-alpha.

PPAR-γ regulates many physiological processes in the kidneys, and its presence in kidney cells ensures its role in normal renal functioning (Corrales et al., 2018). It is expressed by podocytes, glomerular endothelial cells, mesangial cells, proximal convoluted tubules and inner medullary collecting ducts (Figure 2). Kidneys metabolize various substrates, including free fatty acids (FFAs), and PPAR-γ plays a significant role in renal lipid metabolism (Kiss-Tóth and Rőszer, 2009). The FFAs in the glomerular filtrate are reabsorbed in the proximal convoluted tubules and metabolized inside the mitochondria of tubular cells. Therefore, any alteration in the fat metabolism directly affects renal function (Lin and Duann, 2020). A study confirmed that PPAR-γ2 isoform ablation in a leptin-deficient obese ob/ob mouse leads to enhanced renal damage (POKO mouse). The PPAR-γ2 is vital for renal lipid metabolism and insulin sensitivity. It controls the glomerular filtration and proliferation of various fibrotic and inflammatory factors in the glomeruli. PPAR-γ also regulates the expression of adipokine, which plays a renoprotective role by improving insulin sensitivity and inhibiting inflammation (Martínez-García et al., 2015). Another study showed that increased fatty acid levels reduce the PPAR-γ1 expression in the podocytes (Platt and Coward, 2017). Glucose homeostasis is another essential function performed by the kidneys through regulating glucose filtration, reabsorption and gluconeogenesis (Corrales et al., 2018). The renal cortex is the main site for gluconeogenesis; however, glycolysis occurs primarily at the medulla. The glucose reabsorption takes place in the proximal convoluted tubules. It is dependent on sodium-dependent glucose cotransporters (1 and 2) on epithelial cells and glucose transporters (GLUTs) on the basolateral membrane (Szablewski, 2017). The function of these transporters is altered by hyperglycemia; however, PPAR-γ agonism was suggested to maintain the functioning of sodium-dependent glucose cotransporter in diabetic conditions. The kidney also participates in calcium and phosphate metabolism by synthesizing a vital factor, i.e., 1,25 dihydroxy vitamin D3 (Mather and Pollock, 2011). A murine klotho gene expressed in proximal convoluted tubules, distal convoluted tubules and inner medullary collecting ducts is suggested as a target for PPAR-γ. Earlier, this gene was discovered as an antiaging gene, encoding a single-pass transmembrane protein that forms a complex with the FGF receptor and makes a binding site for FGF23. Klotho gene regulates calcitriol’s production and controls urinary calcium, potassium and phosphate excretion. PPAR-γ plays a vital role in mineral metabolism by regulating this gene (Ferrannini et al., 2013). PPAR-γ is expressed by the cells of the juxtaglomerular apparatus, which produce an essential hormone, renin. Renin is a vital element of the renin-angiotensin-aldosterone system (RAAS), regulating the production of angiotensin I from angiotensinogen. Thereby, PPAR-γ regulates the expression of the renin gene and its transcription. PPAR-γ is also a negative regulator of angiotensin II receptor 1 transcription, another vital component of RAAS. The inhibition of the angiotensin II receptor 1 by PPAR-γ activation reduces proteinuria and inflammation in diabetes and hypertension (Corrales et al., 2018). PPAR-γ also plays an essential role in maintaining renal vascular pressure, which is highly expressed in endothelial and smooth muscle cells. Its expression for maintaining blood pressure could be due to stimulating vasoactive factors released by endothelium or downregulating the ANG II type 1 (Sugawara et al., 2010). There is a significant relationship between the renin-angiotensin system (RAS) and PPAR-signaling. TZDs, which are PPAR-γ agonists, cause a reduction in ANG I and II production from human subcutaneous adipocytes and hypertensive rat mesangial cells (Figure 3).

## Synthetic peroxisome proliferator-activated receptor gamma agonists and their limitations

**FIGURE 4:**
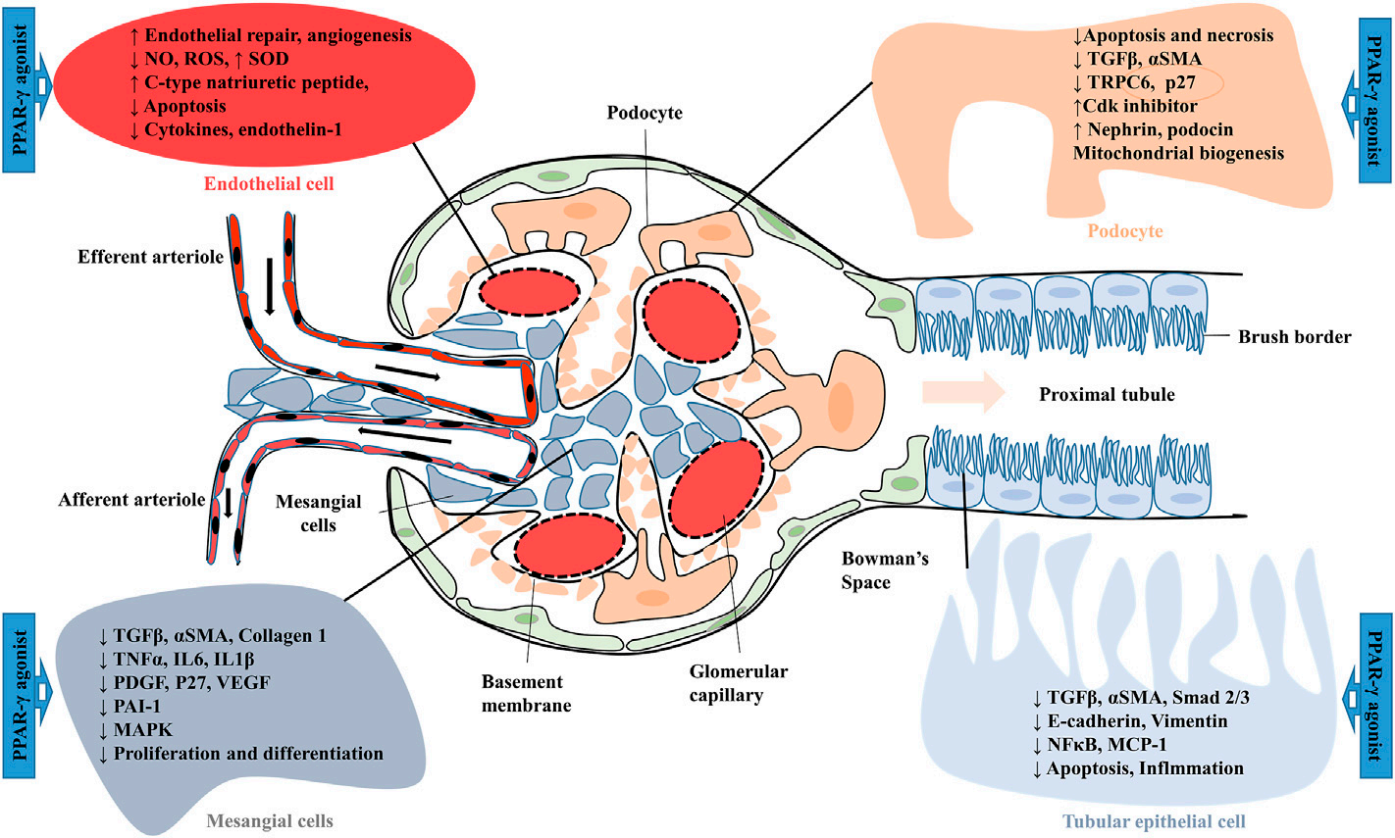
Effect of PPAR-γ agonist on the expression of various factors in glomerular and proximal tubular epithelial cells. MCP 1, Monocyte chemoattractant protein-1; MAPK, mitogen-activated protein kinase; NO, Nitric oxide; NFκB, Nuclear factor-kappa B; PDGF, Platelet-derived growth factor; PAI-1, Plasminogen activator inhibitor-1; ROS, Reactive oxygen species; Smad 2/3, Small mothers against decapentaplegic 2/3; αSMA, Smooth muscle alpha-actin; SOD, Superoxide dismutase; TRPC6, Transient receptor potential cation channel, subfamily C, member 6; TGFβ, Transforming growth factor-beta; VEGF, Vascular endothelial growth factor.

PPAR-γ is a member of one of the most important classes of ligand-activated transcriptional factors, which are expressed in various types of kidney cells. Synthetic agonists of PPAR-γ were suggested to show therapeutic potential in many renal complications (Katoch et al., 2022). Anti-diabetic PPAR-γ agonist pioglitazone was reported effective against renal reperfusion injury. Activation of PPAR-γ directly downregulated the activity of vascularization-related molecules such as nitric oxide (NO), hypoxia-inducible factor -1α (HIF-1α), nitric oxide synthase (eNOS), vascular endothelial growth factor (VEGF) and its receptors (Fetal liver kinase-1 and fms-like-tyrosine kinase-1) (Figure 4). Furthermore, pioglitazone also showed its renoprotective mechanism by increasing antioxidant enzyme (SOD) levels and decreasing lipid peroxidation in rat injured kidneys (Sun et al., 2016). In various rodent models of type-2 diabetes, treatment with thiazolidinedione (TZD) PPAR-γ agonists such as troglitazone, ciglitazone, pioglitazone and rosiglitazone are suggested to improve insulin resistance and glycemic control. These agonists also ameliorate diabetic nephropathy (DN) by inhibiting glomerular hypertrophy, improving proteinuria, circulating lipid levels and renal functions (Zhang and Guan, 2005). The rosiglitazone treatment also prevented podocyte loss, glomerular fibronectin expression, and ROS production in mice with DN. Similarly, the protective mechanism of PPAR-γ agonist on podocyte injury was investigated in puromycin amino nucleoside nephrosis (PAN). Pioglitazone decreases the PAN-mediated podocyte cell injury, necrosis and cell apoptosis by decreasing caspase-3 activity and increasing anti-apoptotic Bcl-XL expression (Kanjanabuch et al., 2007; Ma et al., 2020). Synthetic PPAR-γ agonists such as pioglitazone, troglitazone and rosiglitazone have also shown a blood pressure-lowering effect by inhibiting angiotensin II (Ang II) type receptor expression as well as the Ang II signalling pathway. In a study, male hypertensive Wistar rats treated with rosiglitazone (5 mg/kg/day) decreased blood pressure levels and improved the vascular response to acetylcholine. Serum analysis revealed a lower serum Ang II level and an elevated Ang-(1–7). Rosiglitazone also decreased oxidative stress by lowering 8-hydroxy-2′-deoxyguanosine and malondialdehyde (MDA) levels. Furthermore, rosiglitazone mediated activation of PPAR-γ downregulated RAS protein expression by decreasing ACE and Ang II type 1 receptors and increasing ACE2 and AT2 receptors in male hypertensive rats (Sánchez-Aguilar et al., 2019). The PPAR-γ agonist telmisartan is used to treat hypertension and diabetes. Telmisartan showed a renoprotective effect by inhibiting angiotensin II receptor 1 and decreasing proteinuria and inflammation. The unique property of telmisartan is that the ligand-binding domain of PPAR-γ is different from the site used by TZDs (Benson et al., 2004).

Despite the renoprotective role, the synthetic PPAR-γ agonists such as rosiglitazone, pioglitazone, and troglitazone are also reported to show various adverse effects such as fluid retention or blood volume expansion, obesity, heart disease and hepatotoxicity (Sauer, 2015). These adverse effects might be due to over-activation of PPAR-γ by synthetic drugs at different tissue sites (such as podocytes and collecting duct systems). In the year 2013, FDA restricts the use of rosiglitazone in patients with atherosclerosis (Florez et al., 2015). There is increasing evidence of bone fractures in females medicated with rosiglitazone after menopause. Although, further discussion in the FDA panel in 2013 reviewed all the available trial data and found no significant evidence against cardiovascular complications induced by rosiglitazone treatment (Mitka, 2013). Recent studies also revealed the carcinogenic effects of pioglitazone as a potential risk factor for bladder cancer. Their first indication comes from the clinical trials, which showed more bladder tumours in patients treated with pioglitazone versus placebo (Dangi-Garimella, 2016; Tang et al., 2018).

Similarly, a cohort study compared with various hypoglycemic agents to explore the link between TZD treatment, myocardial infarction and mortality. The study suggested that TZD treatment increased the risk of heart failure even in the young age group. Thus, physicians should use TZDs with caution in patients with heart disease (Lebovitz, 2019). Troglitazone, the first compound approved by the FDA to treat type-2 diabetes, was restricted after individual cases of idiosyncratic hepatotoxicity were reported globally. Ong et al. (2007) suggested that troglitazone did not cause liver injury in normal healthy rodents (wild type), while animals with genetic abnormalities in defence mechanism may be susceptible to its adverse effects. In the study, the heterozygous superoxide dismutase 2 (Sod2+/−) mice were daily administrated with troglitazone for 4 weeks, and the higher dose of troglitazone in mice was equivalent to the human dose. In the wild type, troglitazone did not show any hepatotoxic effects; however, in Sod2+/− mice, elevation in serum aminotransferase activity and hepatocellular necrosis were observed at a higher dose. Moreover, hepatic mitochondria isolated from the troglitazone-treated mice showed an increase in oxidative stress level by decreasing mitochondrial membrane potential, aconitase (by 45%) and complex I activity and increased hydroxy-deoxyguanosine level (Jaeschke, 2007; Ong et al., 2007). Thus, synthetic forms of PPAR-γ with their disease prevention property also have contradictory sides. Therefore, natural PPAR-γ agonists can play an important role in treating renal diseases with minimal side effects.

## Natural peroxisome proliferator-activated receptor gamma agonists

**FIGURE 5:**
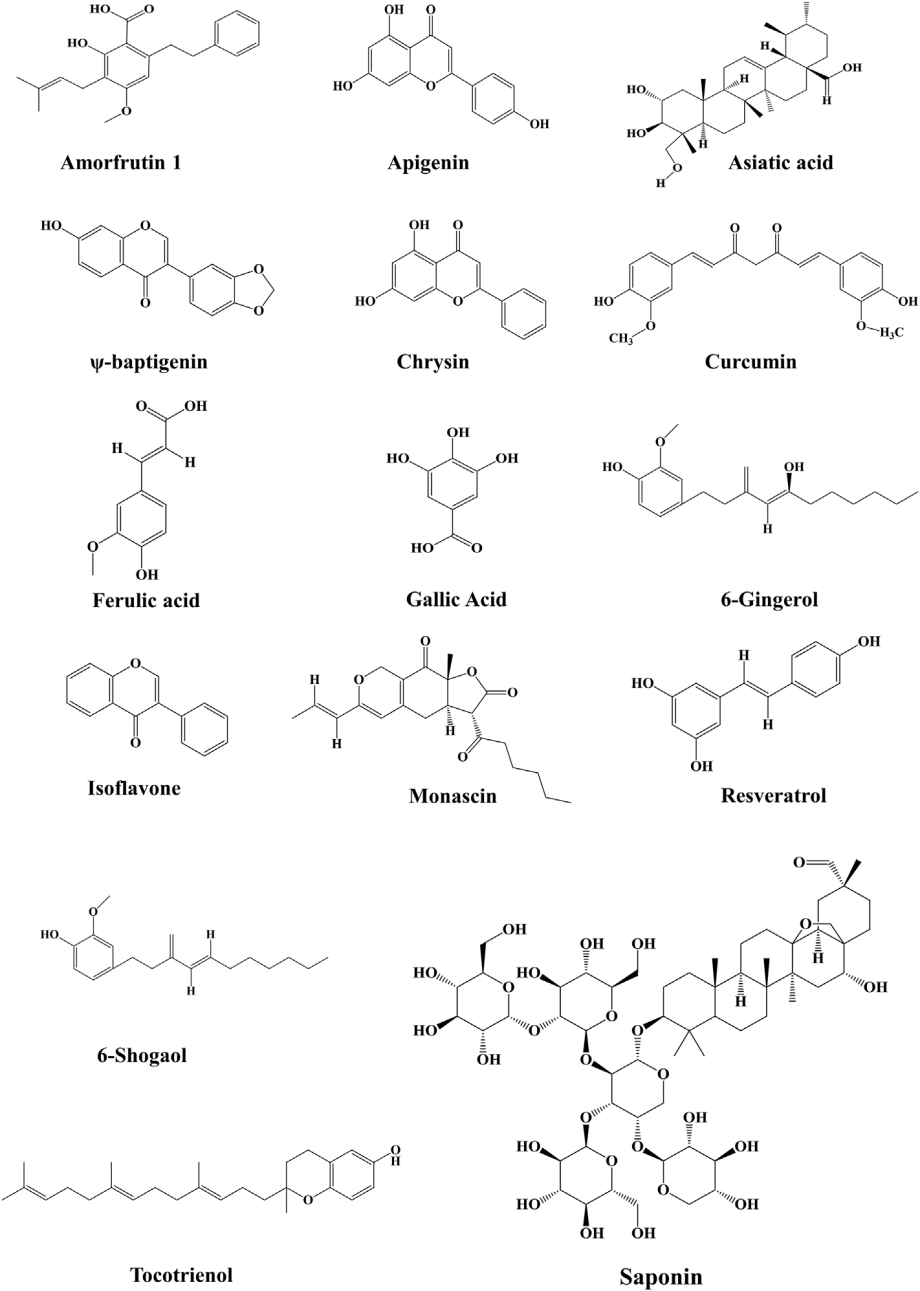
Chemical structures of various natural PPARγ agonists from different sources have shown efficacy against kidney diseases.

The synthetic PPAR-γ agonists manifested many adverse effects due to complete PPAR-γ activation, which led to their removal from the global market in contrast to endogenous PPAR-γ ligands, which are weak agonists. Therefore, researchers are exploring new potential PPAR-γ modulators, which are highly specific in their binding at the active site of PPAR and have minimal side effects (Wang et al., 2014). Medicinal plants or plant-derived bioactive components have high chemical scaffold diversity, making them an ideal source for discovering and developing novel drugs. Several herbal constituents and extracts were identified as PPAR agonists, including dietary polyunsaturated fatty acids (PUFA), flavonoids, isoflavones, lignans, sesquiterpene, amorfrutins and diterpene quinone derivatives (Weidner et al., 2013; Tan et al., 2017). However, the physiological effects of all these natural PPAR-γ agonists are not thoroughly investigated. They need further investigation regarding binding their affinity and role in various kidney diseases. A list of natural PPAR-γ agonists with their effects in experimental models is given in Table 1. The structures of major natural PPAR-γ agonists against kidney disease are given in Figure 5.

**TABLE 1:** Natural PPAR-γ agonists and their effect on kidney cells *in vitro* and *in vivo*.

PPAR-γ agonists	Modal	Route of administration	Dose	Findings	References
Amorfrutin 1	C57BL/6 mice (4 weeks)	Orally in diet	100 mg/kg	Decreases the level of circulating free fatty acids and leptin	Weidner (2013)
Apigenin	Rats (4 weeks)	Orally in diet	0.2%	Treatment significantly attenuated the DOCA-salt-induced conformational and functional damage to the kidney through downregulation of the TGF-β/Smad2/3 pathway and extracellular matrix proteins	Wei et al. (2017), Zhu et al. (2016)
Asiatic acid	UUO rats (7 days)	Orally	10 mg/kg	Treatment showed an antifibrotic mechanism. And increase the level of endogenous PPAR-γ ligand	Zhang et al. (2020)
*Chrysanthemum morifolium* capitulum	HEK293	NA	100 μg/ml	Decrease the hypertensive hypertrophy in SD rats via reducing blood pressure and regulating myocardial energy metabolism	Zhang et al. (2019)
Chrysin	Rats (28 days)	Orally	60 mg/kg	Improve the biochemical alteration, AGE levels and renal dysfunction in diabetic rats	Rani et al. (2016)
Conjugated linoleic acid (CLA)	Mice (28 days)	Orally	1% and 3%	Treatment reduced the deposition of immunoglobulin in glomeruli and showed an anti-inflammatory response by reducing the production of IL-6 and TGF-β.	Hammond, (2015)
Curcumin	Rats (8 weeks)	Orally	80 mg/kg	Treatment significantly improved the insulin sensitivity and biochemical alterations in diabetic mice	Jayakumar et al. (2016)
Fish oil and Ferulic acid	Rats (19 days)	Orally	Fish oil (5 ml/kg) and Ferulic acid 9,100 mg/kg)	Both treatments enhanced the catalase and PPAR-γ gene expression in rat kidneys and showed clearly as renoprotective.	El-Ashmawy et al. (2018)
Gallic acid	Rats (7 days)	Orally	50,100 and 200 mg/kg	Gallic acid significantly ameliorate the renal ischemia perfusion induced biochemical and histopathological alterations	Singh et al. (2014)
Green tea polyphenols	Rats (26 weeks)	Orally in water	400–600 mg/kg	Improved the increased body weight, VAT accumulation and decreased level of circulating adiponectin.	Tian et al. (2013)
Isoflavone	Zuckar rats (Male-8weeks and Female-11 weeks) RAW 264.7 cells (6, 9, 12 and 24 h)	Orally in diet	1.16 g/kg	Significantly reduced the triglyceride, total cholesterol level and proteinuria	Mezei et al. (2003)
Monascin	Balb/c mice (28 days)	Intraperitoneal injection	10 mg/kg	Improving high blood sugar level, oxidative damage and inhibiting PPAR-γ phosphorylation in diabetic mice.	Hsu and Pan, (2014)
*Picrorrhiza kurroa* (Iridoid glycoside rich fraction)	Rats (21 days)	Orally	100 and 200 mg/kg	Treatment showed antihyperglycemic effects and improvement in hepatic and renal function through elevation in antioxidant enzymes level	Sharma et al. (2017)
Resveratrol (RSV)	Zukar rats (6 weeks)	Orally in diet	200 mg/kg and	RSV showed elevation of circulating adiponectin and reduction in albuminuria, mesangial matrix expansion, inflammation and apoptosis	Beaudoin et al. (2013), Park et al. (2016)
*Tinospora cordifolia* (70% ethanolic extract)	Rats (8 weeks)/MES 13 (72 h) and NRK 52E (72 h)	Orally	50, 100 and 200 mg/kg (Rat) and 50, 100 and 200 μg/kg (cells)	Showed anti-inflammatory, anti-fibrotic, activity as well as improve the pathological alterations in renal tissues	Patial et al. (2021)
Tocotrienol	C57BL/6J mice (12 weeks)	Orally	100 and 300 mg/kg	Significantly decrease the oxidative stress, cellular damage and inflammation via downregulating NF-κB, TNF-α and caspases-3 activity	Pang & Chin (2019), Lee & Lim (2018)
Zhen-wu-tang (ZWT)	Rats (16 weeks)	Orally	16.8 g/kg and 1mg/kg	IgA deposition was significantly decreased by both higher and lower doses of ZWT. In addition, reduced levels of Nephrin and podocin, proteins in IgAN were also upregulated by ZWT treatment	Liu et al. (2018)
Ѡ-3 polyunsaturated fatty acid (PUFA)	Human renal tubular cells (24 h)	NA	EPA (10 μmole/L)-DHA (100 μmole/L)	PUFA significantly decreased the lipopolysaccharides (LPS) mediated NF-κB activation and MCP-1 expression.	Li et al. (2005)

AGE, advanced glycation end products; BAEC, bovine aorta endothelial cells; COX-2, Cyclooxygenase-2; Caspase-3, cysteine-aspartic proteases-3; DHA: docosahexaenoic acid; DOCA, deoxycorticosterone acetate; EPA, eicosapentaenoic acid; GLUT-4, glucose transporter type 4; HEK-293, cells, Human embryonic kidney 293 cells; IgAN, immunoglobulin A nephropathy; IL-6, interleukin-6; MCP-1, monocyte chemoattractant protein-1; NF-кB, nuclear factor kappa-light-chain-enhancer of activated B cells; TGFβ, transforming growth factor-beta; TNFα, tumor necrosis factor-alpha; VAT, visceral adipose tissue.

### Peroxisome proliferator-activated receptor gamma natural agonists in acute kidney injury

Acute kidney injury (AKI) is characterized by a sudden loss of renal function, which generally develops within a week. This could be caused due to ischemia, the toxicity of drugs/hazardous compounds, severe inflammation and pathologic occlusion of the urinary tract. AKI incidence is about 4–20% and increases by 10% annually. The pathologies of AKI include retention of nitrogenous waste, body fluid imbalance and metabolic acidosis (Wu et al., 2019). Ischemia-reperfusion injury (IRI) is one of the major causes of AKI, leading to vascular endothelial and renal tubular cell dysfunction. IRI affects the mitochondrial function and depletes the cell’s adenosine triphosphate (ATP). This accounts for the depletion of ATP membrane ion pumps and rises in cytosolic calcium levels resulting in cell death. This further led to the release of proinflammatory cytokines such as IL-1, 6, 8, tumour TNF-α, nuclear factor kappa B (NF-κB) and monocyte chemoattractant protein-1 (MCP-1), resulting in permanent renal damage (Abdelrahman et al., 2005). PPAR-γ agonists potentially downregulate the proinflammatory cytokines released during the differentiation of macrophages. The gallic acid (Figure 5) was reported to activate PPAR-γ and prevent IRI by inhibiting the expression of inducible nitric oxide synthase, MCP-1, ICAM-1 and proinflammatory cytokines (Singh et al., 2014). Similarly, in one of our studies, iridoid glycosides from *Picrorrhiza kurroa* attenuated the cyclophosphamide-induced acute renal damage by activating PPAR-γ. Cyclophosphamide is an anticancer drug, and acute renal injury is one of its side effects. The compound leads to elevated renal injury markers, tubular damage, apoptosis and renal inflammation. However, the PPAR-γ activation by the iridoid glycosides resulted in improved renal function and pathology, reduced inflammation and caspase 3/9 activity (Sharma et al., 2017). In both the above studies, the role of PPAR-γ was confirmed by the use of PPAR-γ antagonist bisphenol A diglycidyl ether (BADGE), which inhibited the protective effect of gallic acid and iridoid glycosides. Gentamicin is an aminoglycoside antibiotic used to treat infections related to gram-negative bacteria. The major drawbacks of its use are nephrotoxicity and ototoxicity. The partial up takes of gentamicin by the proximal convoluted tubules elevate its concentration in the tubular cells and cause renal damage. The gentamicin generates the reactive oxygen species, resulting in the altered synthesis of macromolecules, disturbed cell function and cell death. It further leads to mesangial and vascular impairments and the release of proinflammatory cytokines (Tavafi, 2012).

In a study, fish oil and ferulic acid showed a protective effect against gentamicin-induced renal damage by upregulation of the PPAR-γ gene. The PPAR-γ activation mediated the antioxidant, anti-inflammatory and other renoprotective effects in gentamicin-induced toxicity (El-Ashmawy et al., 2018). Asiatic acid (AA) is a pentacyclic triterpenoid present in *Centella asiatica* known for its several therapeutic effects (Figure 5) (James and Dubery, 2009). Zhang et al. (2020) evaluated the anti-fibrotic potential of AA in unilateral ureteral occlusion (UUO) induced renal damage. UUO led to ROS generation, oxidative damage, renal dysfunction and renal fibrosis by activating of TGF-β/SMAD pathway. However, AA treatment effectively ameliorated oxidative damage in renal tissue. Interestingly the increased levels of endogenous PPAR-γ ligand 15d-PGJ2 in plasma in AA-treated groups indicated the role of PPAR-γ in the anti-fibrotic mechanism of AA (Zhang et al., 2020). In a study, lipopolysaccharides (LPS) exposed to immortalized human proximal tubular cell line (HK-2) were treated with Ѡ-3 polyunsaturated fatty acid (PUFA) containing eicosapentaenoic acid (EPA; 10 μmole/L) and docosahexaenoic acid (DHA; 100 μmole/L). The compounds significantly decreased LPS-mediated NF-κB activation and monocyte chemoattractant protein expression (MCP-1). However, inhibition of PPAR-γ in HK-2 cells with bisphenol A diglycidyl ether (BADGE) reversed the inhibitory effect of PUFA on MCP-1 and NF-κB activation. Moreover, overexpression of PPAR-γ in HK-2 cells by transient transfection further suppressed the NF-κB activation, indicating the role of PPAR-γ in the protective effect of PUFA (Li et al., 2005).

### Peroxisome proliferator-activated receptor gamma natural agonists in diabetic kidney disease

Diabetic kidney disease (DKD) is one of the most common microvascular complications of both type 1 (T1DM) and type 2 diabetes mellitus (T2DM) and the leading cause of the end-stage renal disease (ESRD) worldwide. (Zheng et al., 2021). A cross-section study conducted in different regions of the world reported that around 8% (approx. 350 million) of the population suffer from diabetes, and greater than 40% of diabetic patients will develop kidney disease by the year 2035 (Gheith et al., 2016). WHO established a global action plan to reduce the impact of four non-communicable diseases: cancer, heart disease, chronic respiratory disease and diabetes (Luyckx et al., 2018). The clinical indications of DKD include elevation in glomerular filtration rate (GFR), high blood pressure, proteinuria (or albuminuria rate >300mg/dl), increased levels of circulating lipids and dyslipidaemia (Lim, 2014). The epigenetic factors involved in the pathogenesis of DKD are DNA methylation, modulation of ribonucleic acids and alteration in histone proteins. Modern interventions overthrowing this pathological condition include regulating blood pressure by inhibiting angiotensin-II converting enzymes and reducing proteins and salt concentration (Fountain and Lappin, 2017).

Resveratrol (RSV) is a polyphenolic compound having anti-diabetic properties and a PPAR-γ activating effect (Figure 5). In diabetic kidney disease, resveratrol showed protective effects by improving the levels of antioxidant enzymes such as superoxide dismutase (SOD), glutathione peroxidase (GPx), catalase (CAT) and levels of vitamin C and E. Further, RSV treatment upregulated the level of NrF2 and its downstream targets (glutamyl cysteine synthetase, heme oxygenase-1) in the renal tissue (Li et al., 2005; Castrejón-Tellez et al., 2016). Kitada et al. (2011) reported that resveratrol treatment (5 mg or 10 mg/kg) improved ROS generation, renal oxidative stress, urinary protein excretion and renal dysfunction in diabetic rats. The effect of RSV on mitochondrial content and respiration glyceroneogenesis was also assessed in Zucker diabetic fatty (ZDF) rats. Rats fed with a chow diet and RSV showed an alleviated level of circulating adiponectin, lower glucose and insulin tolerance in contrast to the normal control (Beaudoin et al., 2013). Another study revealed the protective effect of RSV in post-contrast acute kidney injury in rabbits after diabetic nephropathy. RSV treatment increased the expressions of peroxisome proliferator-activated receptor-gamma coactivator-1 alpha (PGC-1α) and reduced mitochondrial dysfunction, renal hypoxia, and renal tubular cell apoptosis (Wang et al., 2019). Similarly, Park et al. (2016) reported that RSV improved serum adiponectin levels and reduced albuminuria. Further, RSV reduced the mesangial matrix expansion, inflammation and apoptosis in the glomeruli by modulating the expression of PGC-1α, phosphorylated acetyl-CoA carboxylase estrogen-related receptor-1α, and sterol regulatory element-binding protein 1 (Park et al., 2016). There are limited studies on the meaningful effect of RSV in humans with kidney disease. RSV administration (450 mg/day) for 12 weeks in peritoneal dialysis patients resulted in a significant improvement in mean net ultrafiltration volume and rate *via.* improving angiogenesis markers such as VEGF, fetal liver kinase-1 and adiponectin levels (Lin et al., 2016).

Vitamin E (Vit E) is a fat-soluble vitamin which includes two compounds, tocopherol and tocotrienols (T3). Tocotrienol potentially inhibits oxidative stress, cellular damage and inflammation by downregulating NF-κB, TNF-α and caspases-3 activity. Tocotrienol inhibits the process of adipogenesis by upregulation of enzymes/proteins involved in lipid metabolisms such as carnitine palmitoyltransferase, cytochrome p450 and PPAR α/δ (Lee and Lim, 2018). A lower concentration of tocotrienol (0.024–2.4 µM) suppresses the upregulation of TNF-α, MCP-1 and IL-6 expression with the restoration of adiponectin and PPAR γ expression in 3T3-L1 cells. In addition, histological alterations such as tubular and glomerular damage, inflammation and mesangial expansion were significantly reduced by rice bran oil enriched with tocotrienol (Fang et al., 2010; Pang and Chin, 2019). Curcumin is suggested to have an anti-diabetic role and is also reported to activate PPAR-γ. In a study, diabetic rats were co-administrated with curcumin for 8 weeks (80 mg/kg BW) showed improved levels of LDL, triglycerides, total cholesterol, creatinine, urea and proteinuria. Curcumin also improved lipid peroxidation and antioxidant enzyme levels in liver and kidney tissues. Further, molecular docking studies of curcumin as a PPAR-γ agonist showed a relatively high binding affinity of curcumin with PPAR-γ in comparison to pioglitazone. In addition, histopathological alterations in the liver and kidney of diabetic rats were reduced by curcumin, which provides new insight regarding curcumin as a PPAR-γ agonist for the treatment of insulin sensitivity and diabetes-related complications (Jayakumar et al., 2016). Weir et al. measured the effect of micro-particle curcumin on two important markers of CKD progression: albuminuria and estimated glomerular filtration rate (eGFR). Participants were randomly assigned to receive micro-particle curcumin 90 mg once daily or a matching placebo for 6 months. As we mentioned above, due to its antioxidant and anti-inflammatory potential micro-particle curcumin showed promising results in the treatment of CKD (Weir et al., 2018; Nowak et al., 2022)

Advanced glycation end products (AGEs) play a significant role in the generation of ROS and proinflammatory cytokines in diabetes, ultimately leading to renal damage. RAGE-AGE activation in the kidney causes fibrotic response via activating intracellular pathways such as NF-кB, MAPK/ERK and phosphatidylinositol 3 kinase/protein kinase B (PI3K/Akt) pathway (Nowotny et al., 2015). A natural flavonoid, Chrysin was shown to improve the biochemical alteration, AGE levels and renal dysfunction in diabetic rats (Figure 5). Chrysin treatment significantly reduced the RAGE expression and promoted PPAR-γ activation in diabetic rats. In addition, chrysin improved the antioxidant enzyme level (GSH, CAT and MnSOD) and decreased inflammation *via* inhibiting NF-κB/IKK expression. Thus, PPAR-γ activation directly inhibits AGE-RAGE-mediated oxidative damage and inflammation in diabetic rats (Rani et al., 2016). Similarly, Monascus species contain some active bioactive yellow pigments with anti-inflammatory properties such as monascin and ankaflavin. Monascin is potentially known to improve elevated triglycerides, total cholesterol, low-density lipoproteins (LDL) and diabetes. However, it also enhances the high-density lipoprotein level (HDL) and possesses preventive effects against the fatty liver (Lee et al., 2013). Monascin is a natural PPAR-γ agonist which showed an anti-inflammatory effect mediated by inhibition of c-Jun terminal kinase (JNK), extracellular signal-regulated kinase (ERK), p38 kinase and cytokines production, as well as anti-diabetic effect via improving high blood sugar level inhibits insulin resistance and reducing oxidative stress. Monascin was also shown to inhibit PPAR-γ phosphorylation in methyl glyoxal treated Balb/c mice and RIN-m5F cells (cells which secrete and produce insulin), attenuating insulin expression and reducing the blood glucose level in mice fed with a fructose-rich diet (Hsu and Pan, 2014). Amorfrutin is a member of the phenyl terpenoids family with a glucose-lowering capacity and is used as a traditional herb for its anti-inflammatory properties. The small, lipophilic amorfrutin class consists of a 2-hydroxybenzoic acid core structure decorated with phenyl and isoprenyl moieties (Figure 5). It potentially inhibits NFкB by TNFα mediated IкBα degradation. Amorfrutin B is a selective modulator of PPAR-γ isolated from the edible fruits of *Amorpha fruticosa* and the roots of *Glycyrrhiza foetida.* The binding affinity of amorfrutin B at PPAR-γ is comparable to its other isoforms, PPAR-α and PPAR-β/δ. After an interval of amortrutin B treatment in diabetic mice, there is considerable improvement in insulin sensitivity, impaired glucose tolerance and blood lipid profile (Weidner et al., 2013). Our recent study evaluated the protective effect of *Tinospora cordifolia* stem extract against diabetic nephropathy. The various phytoconstituents present in the extract were found to show high binding affinity with the PPAR-γ *in silico*. *In vitro*, the extract activated the PPAR-γ expression in the high glucose treated mesangial (MES 13) and tubular (NRK 52E) cells. Further, the extract treatment in diabetic rats prevented the deterioration of renal function and reduced the pathological changes in the renal tissues. The upregulated PPAR-γ expression in the kidney tissue of the extract-treated group suggested the anti-inflammatory and antifibrotic effects in diabetic rats were due to the activation of PPAR-γ (Patial et al., 2021).

### Peroxisome proliferator-activated receptor gamma natural agonists in obesity-related nephropathy

According to the WHO, the frequency of obesity has drastically increased in the last few decades and appealing a serious social-economic problem. Obesity is not only a risk factor for kidney damage, but also increases the severity of preexisting renal injury (Tsuboi et al., 2017). Obesity-related glomerulopathy (ORG) is a common renal condition secondary to obesity, and its detrimental effects are mediated by downstream comorbid conditions such as hyperglycemia or hypertension. Obesity alters the activity of adipokines (adiponectin, leptin, resistin and visfatin), leading to oxidative stress, inflammation, ER stress (by protein unfolding), abnormal fatty acid metabolism, insulin resistance and activation of the rennin-angiotensin-aldosterone system (RAAS) (Al-Suhaimi and Shehzad, 2013). Lipotoxicity is very common in obese people, directly promoting lipogenesis and alleviating the protein level involved in fatty acid oxidation (Unger et al., 2010). The excess fatty acid stored gets converted into lipid droplets by esterification, which further causes renal cell impairment (especially podocytes), and cell death contributing to CKD (Wahl et al., 2016). PPAR-γ plays an important role in lipid metabolism which, suggests its direct link with obesity and kidney damage (Escasany et al., 2019). Kume et al. (2007) reported the link between renal lipid metabolism and kidney injury in insulin-sensitive heterozygous PPAR-γ deficient mice. Wild mice fed with a high-fat diet for 16 weeks induced subsequent lipid droplet accumulation, abnormal fatty acid metabolism and progressive loss of renal function, including glomerulopathy and RAAS activation. The reduced level of lipogenic enzymes (fatty acid synthase and acetyl CoA carboxylase) and their mRNA level suggested increased renal lipogenesis. However, the mRNA expression of enzymes responsible for fatty acid oxidation (Carnitine palmitoyl Acyl-CoA transferase) was found to be downregulated (Kume et al., 2007). A study showed that palmitic acid treatment in mouse podocytes induces lipogenesis in the cells, further promoting glomerulopathy, inflammation and oxidative stress. Further, palmitic acid treatment reduced the RNA levels of PPAR-α in the podocytes, whereas PPAR-γ levels remain undetectable (Marcia-Fracia et al., 2015). An adipocytokine, adiponectin, secrets from the fat tissue in the bloodstream and contributes 0.01% of total plasma proteins. Obesity causes elevated levels of adiponectin in the bloodstream. The factors like PPAR-γ, kruppel-like factor 7 (KLF7) and sterol regulator binding protein-1 (SREBP-1) are the regulators of adiponectin. Among all these, PPAR-γ is a key regulator of adiponectin, which enhances its expression by facilitating PPAR-γ binding to the adiponectin promoter. Adiponectin is a downstream target gene of PPAR-γ, while it is phosphorylated from (pPPAR-γ) show inhibitory effects on its expression (Yamauchi et al., 2001).

Green tea polyphenols (GTP) are the potential bioactive components well known for their anti-obesity properties (Bose et al., 2008; Tian et al., 2013). GTP treatment of high-fat diet-fed rats improved the body weight, visceral adipose tissue accumulation and decreased the levels of circulating adiponectin. In the high-fat group, mRNA and protein expression analysis revealed a decreased level of PPAR-γ and an increased level of pPPAR-γ; however, GTP treatment significantly attenuated these alterations. In addition, GTP treatment also inhibited extracellular signal-regulated protein kinase (erk1/2) activation, which might have regulatory effects on the downregulation of PPAR-γ by promoting PPAR-γ phosphorylation (Tian et al., 2013). Borges et al. investigated the effect of GTP on residual albuminuria of diabetic subjects with nephropathy. Patients were assigned to two groups, one receiving GTP (containing 800 mg of epigallocatechin gallate) and the other a placebo group. Treatment with GTP showed a significant reduction in the urinary albumin-creatinine ratio (UACR) by 41%. In addition, *in vitro* studies showed a significant reduction in podocyte apoptosis through the activation of the WNT pathway (Borges et al., 2016). Amorfrutin is a selective modulator of PPAR-γ isolated from the edible fruits of *Amorpha fruticosa* and roots of *Glycyrrhiza fortida*. The protective mechanism of amorfrutin 1 was investigated in C57BL/6 mice fed with a high-fat diet (HFD) for 15 weeks. Early treatment Amorfrutin 1 (A1 + HFA) with HFD significantly improved the HFD-induced weight gain without altering dietary intake. The amorfrutin administration significantly improved the concentration of circulating free fatty acids and elevated the adipose-derived hormone leptin level. In contrast to the synthetic drug rosiglitazone, amorfrutin 1 decreased triglycerides level, insulin resistance, and glucose intolerance in HFD-fed C57BL/6 mice (Weidner et al., 2012).

### Peroxisome proliferator-activated receptor gamma natural agonists in hypertension nephropathy

Hypertension nephropathy most commonly occurs in patients having hypertension and diabetes mellitus. The severity of hypertension determines the risk of renal damage. Hypertension nephrosclerosis commonly manifests as a decreased level of renal function, microalbuminuria and ESRD (Van Buren and Toto, 2011). A series of determinants such as ROS accumulation, RAAS activation and nitric oxide reduction conspire to cause hypertension. RAAS activation and hypervolemia play a pivotal role in increasing blood pressure. The majority of the RAAS elements are present in the kidney, and the intrarenal formation of angiotensin II, regulates blood pressure through vasoconstriction, sodium reabsorption and glomerular hemodynamics. In addition, it also triggers a number of inflammatory and fibrotic pathways (Siragy and Carey, 2010). Thus, the therapeutic interventions which control RAAS activation and decrease renal injury can play an important role against hypertension nephropathy.

Chrysanthemum flowers (*Chrysanthemum morifolium*) are widely present in different parts of the globe and are mostly consumed as herbal tea due to antibacterial, antiviral and immunomodulatory effects. The active components of *C. morifolium* capitulum (MCM) isolated from methanolic extract are determined for its PPAR-γ agonistic activity in a series of concentrations. MCM showed comparable PPAR-γ agonistic activity to pioglitazone (PPAR-γ standard agonist) 100 μg/ml. MCM as an antihypertensive decreased myocardial hypertrophy in SD rats by reducing blood pressure and regulating myocardial energy metabolism. In addition, it further downregulated the expression of HIF-1α and GLUT-4 and upregulated PPAR-α expression (Zhang et al., 2019). Similarly, apigenin (4’,5,7-trihydroxyflavone), a natural flavonoid, has been reported to show antihypertensive activity by downregulating HIF-1α and upregulating PPAR-γ expression (Figure 5). Apigenin also showed protective effects on angiogenesis, hypertension and cystic fibrosis (Zhu et al., 2016). In another study, the anti-hypertensive effect of apigenin was evaluated in deoxycorticosterone acetate (DOCA) salt-induced hypertension in male SD rats. Apigenin treatment for 4 weeks significantly attenuated the DOCA-salt-induced conformational and functional damage to the kidney through the downregulation of the TGF-β/Smad2/3 pathway and extracellular matrix proteins (Wei et al., 2017). Furthermore, apigenin directly activates the transient receptor potential vanilloid 4 (TRPV4) channels and maintains Ca^2+^ homeostasis in hypertensive rats (Wei et al., 2012; Wei et al., 2017).

### Peroxisome proliferator-activated receptor gamma natural agonists in immunoglobulin A (IgA)/autoimmune nephropathy

IgA nephropathy (IgAN) is a common type of glomerulonephritis, an autoimmune disease wherein the subclass of IgA, i.e. IgA1 with galactose-deficient O-glycan (auto-antigen) and anti-glycan deposits in the glomerular mesangium (Rajasekaran et al., 2021). It can occur in individuals of any age group, even at a young age and is primarily known as Berger’s disease. In India, the prevalence of IgAN varies from 7–16%, whereas it varies from 2–52% of all renal biopsies worldwide. Clinical signs of IgA in the kidney include blood and sometimes proteins in urea. In its “classic” overview, IgAN is permeated by a mesangiopathic process that includes the enlargement of the mesangial matrix, mesangial cell proliferation and immunofluorescence assay of IgA (Chacko, 2011). Atherosclerotic metabolic factors, such as triglycerides level, insulin resistance, obesity and elevated uric acid, are also associated with IgAN. PPAR-γ plays a vital role in the pathophysiological condition of IgAN. PPAR-γ activation inhibits the proliferation of mesangial cells, serine protease activity and TGF-β expression, which prevents the progression of glomerulosclerosis. It also affects the immunological machinery involved in IgAN-induced renal injury by altering the function of immune cells such as B-cells, T-cells and macrophages (Ouyang et al., 2016). PPAR-γ showed beneficial effects by reducing IgAN-induced tubule-interstitial inflammation in human proximal tubular epithelial cells (PTEC). PTE cells were treated with a higher concentration of rosiglitazone (0–5 µM), a PPAR- γ agonist, followed by the incubation with IgA-human mesangial cells isolated from IgAN patients. The study showed the downregulation in elevated expression of inflammatory markers such as interleukin-6 (IL-6), NF-κB, and angiotensin II type 1 receptor (ATR1) by PPAR-γ agonist. However, a significant decrease in the expression of ATR1were observed due to inhibition of ERK1/2 activation at the kinase level (Xiao et al., 2009). Lai et al. (2011) studied the individual and combined effects of rosiglitazone and losartan in IgAN-induced rats. IgAN showed elevated expression of TGF-β, ATR1 and intercellular adhesion molecules (ICAM 1), which ultimately results in hematuria, mesangial cell expansion, IgA deposition, glomerulosclerosis and tubule interstitial infiltration of CD25^+^ leukocytes. Treatment with rosiglitazone and losartan effectively decreased the renal expression of proliferating cell nuclear antigen (PCNA), TGF-β, ATR1 and ICAM1 in IgAN rats. Combined treatment decreased the inflammatory cytokines and showed advancement in glomerular and tubular damage by improving glomerular hypercellularity (Lai et al., 2011).

Traditional Chinese medicine, Zhen-wu-tang (ZWT), is widely used to treat various CKD by improving renal function, oxidative damage, and inflammation in kidney cells. ZWT is a combination of five traditional herbs, *Aconitum carmichaelii* Debeaux, *Poria cocos* Wolf, *Atractylodes macrocephala* Koidz, *Paeonia lactiflora* Pall, and *Zingiber offcinale* Roscoe, with disease prevention properties. Several animal studies demonstrated that ZWT ameliorates renal function by downregulating the hyper-activation of RAS and upregulating nephrin and podocin (filtration slits of podocytes) expression in kidneys (Cai et al., 2010). Liu et al. (2018) investigated the therapeutic role of ZWT on podocyte injury in IgAN rats. Rats treated with a higher dose (ZWT-H) and a lower dose (ZWT-L) significantly decreased the proteinuria and urinary red blood cells in the IgAN model compared to the naïve group. The study revealed that ZWT treatment significantly elevated the expression of PPAR-γ and downregulated the higher level of NF-κB and p-IκBα, confirming its PPAR-γ agonistic activity. There was a high deposition of IgA and C3 in glomeruli in IgAN rats, whereas these depositions were significantly decreased by ZWT treatment. In addition, alleviated levels of nephrin and podocin proteins in IgAN were upregulated by ZWT treatment, indicating its protective role against podocyte injury (Liu et al., 2018). Similarly, conjugated linoleic acid (CLA) is a natural PPAR-γ agonist derived from linoleic acid, naturally present in animal-derived foods. CLA showed anti-inflammatory properties due to its biologically active isomers such as trans-10, cis-12 and cis-9, and trans-11 (also known as rumenic acid). The protective mechanism of CLA was studied against autoimmune glomerulonephritis in a murine model and mesangial cells. CLA treatment reduced the deposition of immunoglobulin in glomeruli and showed an anti-inflammatory response by reducing the production of IL-6 and TGF-β. However, in terms of body weight gain and proteinuria, the results were insignificant compared to the control group fed with a standard diet (Hammond, 2015).

## Conclusion and future prospective

Renal disease is the most common disease in the growing population, which makes a global burden of around 8–16%. The most common factors responsible for kidney diseases are diabetes, hypertension, obesity and autoimmune disease. However, most people who need treatments are not survived due to lacking renal replacement therapy. The most effective methods which may help to overcome the progression of renal disease in early recognition, specific drug therapy and risk management. Research efforts have been made to explore therapeutic factors such as PPAR, which give a promising contribution to overcoming this situation. PPAR-γ is a ligand-activated superfamily member of ligand-dependent transcription and modulates numerous biological processes, such as lipolysis, diabetes, cancer, cell differentiation and glycolysis. It is primarily expressed in adipose tissue and is also present in several kidney cells. The therapeutic role of PPAR-γ is not only due to the regulation of glucose and lipid metabolism, but it also shows potential as an anti-inflammatory factor *via* inhibiting cytokine production. There are several PPAR-γ agonists which significantly improve clinical outcomes of kidney diseases. The agonists like troglitazone, ciglitazone, pioglitazone and rosiglitazone improve insulin resistance, glycaemic control, glomerular hypertrophy, proteinuria, circulating lipid level and renal function in the kidney. However, despite their reno-protective effect, these compounds also cause many adverse effects on human health, such as fluid retention or blood volume expansion, obesity, heart disease and hepatotoxicity. Thererfore, researchers are trying to explore natural agents, which are highly specific in terms of their binding affinity with PPAR. Natural compounds such as dietary polyunsaturated fatty acids (PUFA), flavonoids, isoflavones, lignans, sesquiterpene, amorfrutins and diterpene quinone derivatives are potentially known for their activity as PPAR-γ agonists and did not show any adverse effects such as weight gain, fluid retention and fracture risk. However, the real future challenge with these natural compounds is to elucidate the mechanism of action in order to validate their therapeutic potential against renal disease.

## Glossary

AA: Asiatic Acid
ACR-2: Angiotensin-converting enzyme 2
AF: Activation function
AKI: Acute kidney injury
ANG-II: Angiotensin II;
AT1: angiotensin type 1 receptor
ATP: Adenosine triphosphate;
ATR1: Angiotensin II type 1 receptor
BADGE: Bisphenol A diglycidyl ether
Bax: BCL2-associated X protein
BcL-2: B-cell leukemia/ lymphoma 2
BSA: Bovine serum albumin
Caspase-3: Cysteine-aspartic proteases-3;
CAT: Catalase;
CdK: Cyclin-dependent kinase
CKD: Chronic kidney disease
CLA: Conjugated linoleic acid
COX-2: Cyclooxygenase-2
DBD: DNA-binding domain
DHA: Docosahexaenoic acid
DKD: Diabetic kidney disease
DN: Diabetic nephropathy
DOCA: Deoxycorticosterone acetate
eNOS: Nitric oxide synthase
EPA: Eicosapentaenoic acid
ERK: extracellular signal-regulated kinase
ESKD: End stage kidney disease
ESRD: End-Stage Renal Disease
FDA: Food and drug administration
FFA: Free fatty acid;
FGF: Fibroblast growth factor
GLUT: Glucose transporter
GPx: Glutathione peroxidase
GTP: Green tea polyphenols
HDL: High density lipoprotein
HFD: High fat diet
HIFα1: Hypoxia inducible factor-1alpha
ICAM: Intercellular adhesion molecules
ICAM-1: Intercellular adhesion molecule 1
IgAN: Immunoglobulin A nephropathy
IL: Interleukin
IRI: Ischemia reperfusion injury
IκBα: Nuclear factor of kappa light polypeptide gene enhancer in B-cells inhibitor alpha
JNK: c-Jun terminal kinase
KLF7: kruppel-like factor 7
LBD: ligand-binding domain
LDL: Low density lipoprotein;
LPS: Lipopolysaccharides;
MAPK: Mitogen-activated protein kinase
MCP-1: Monocyte chemoattractant protein-1
MDA: Malondialdehyde
mRNA: Messenger ribonucleic acid
NFκB: Nuclear factor kappa- B
NO: Nitric oxide
ORG: Obesity related glomerulopathy
PAI-1: Plasminogen activator inhibitor-1
PAN: Puromycin amino nucleoside nephrosis
PCNA: Proliferating cell nuclear antigen
PGC-1: Peroxisome proliferator-activated receptor-gamma coactivator-1
PPAR-γ: Peroxisome proliferator- activated receptor gamma
PPRE: Peroxisome proliferator-activated receptor-gamma response element
PTEC: Proximal tubular epithelial cells
PUFA: Polyunsaturated fatty acid
RAAS: Rennin-angiotensin-aldosterone system
AGEs: Advanced glycation end products
RAGE: Receptor for AGEs (RAGE)
RAS: Renin angiotensin system
ROS: Reactive oxygen species
RSV: Resveratrol
RXR: Retinoid X receptor
SMAD: Small mothers against decapentaplegic
SOD: Superoxide dismutase
SRC: Steroid receptor coactivator
SREBP: sterol regulator bonding protein-1c
STAT: Signal transducer and activator of transcription
T1DM: Type 1 diabetes mellitus
T2DM: Type 2 diabetes mellitus
TBARS: Thiobarbituric acid reactive substances
TGFβ: Transforming growth factor-beta
TNFα: Tumor necrosis factor-alpha
TRPV4: Transient receptor potential vanilloid 4
TZD: Thiazolidinediones
UUO: Unilateral ureteral occlusion
VAT: Visceral adipose tissue
VEGF: Vascular endothelial growth factor
WHO: World health organization
ZDF: Zucker diabetic fatty
ZWT: Zhen-wu-tang
αSMA: Smooth muscle alpha-actin
